# Biomass-degrading glycoside hydrolases of archaeal origin

**DOI:** 10.1186/s13068-020-01792-y

**Published:** 2020-09-02

**Authors:** Marcel Suleiman, Anna Krüger, Garabed Antranikian

**Affiliations:** 1 Institute of Technical Microbiology, University of Technology Hamburg, Hamburg, Germany; 2 Department of Evolutionary Biology and Environmental Studies, University of Zurich, Zurich, Switzerland

**Keywords:** Archaea, Glycoside hydrolases, Hyperthermozymes, Hydrothermal systems, Bioeconomy

## Abstract

During the last decades, the impact of hyperthermophiles and their enzymes has been intensively investigated for implementation in various high-temperature biotechnological processes. Biocatalysts of hyperthermophiles have proven to show extremely high thermo-activities and thermo-stabilities and are identified as suitable candidates for numerous industrial processes with harsh conditions, including the process of an efficient plant biomass pretreatment and conversion. Already-characterized archaea-originated glycoside hydrolases (GHs) have shown highly impressive features and numerous enzyme characterizations indicated that these biocatalysts show maximum activities at a higher temperature range compared to bacterial ones. However, compared to bacterial biomass-degrading enzymes, the number of characterized archaeal ones remains low. To discover new promising archaeal GH candidates, it is necessary to study in detail the microbiology and enzymology of extremely high-temperature habitats, ranging from terrestrial to marine hydrothermal systems. State-of-the art technologies such as sequencing of genomes and metagenomes and automated binning of genomes out of metagenomes, combined with classical microbiological culture-dependent approaches, have been successfully performed to detect novel promising biomass-degrading hyperthermozymes. In this review, we will focus on the detection, characterization and similarities of archaeal GHs and their unique characteristics. The potential of hyperthermozymes and their impact on high-temperature industrial applications have not yet been exhausted.

## Background

**Fig. 1:**
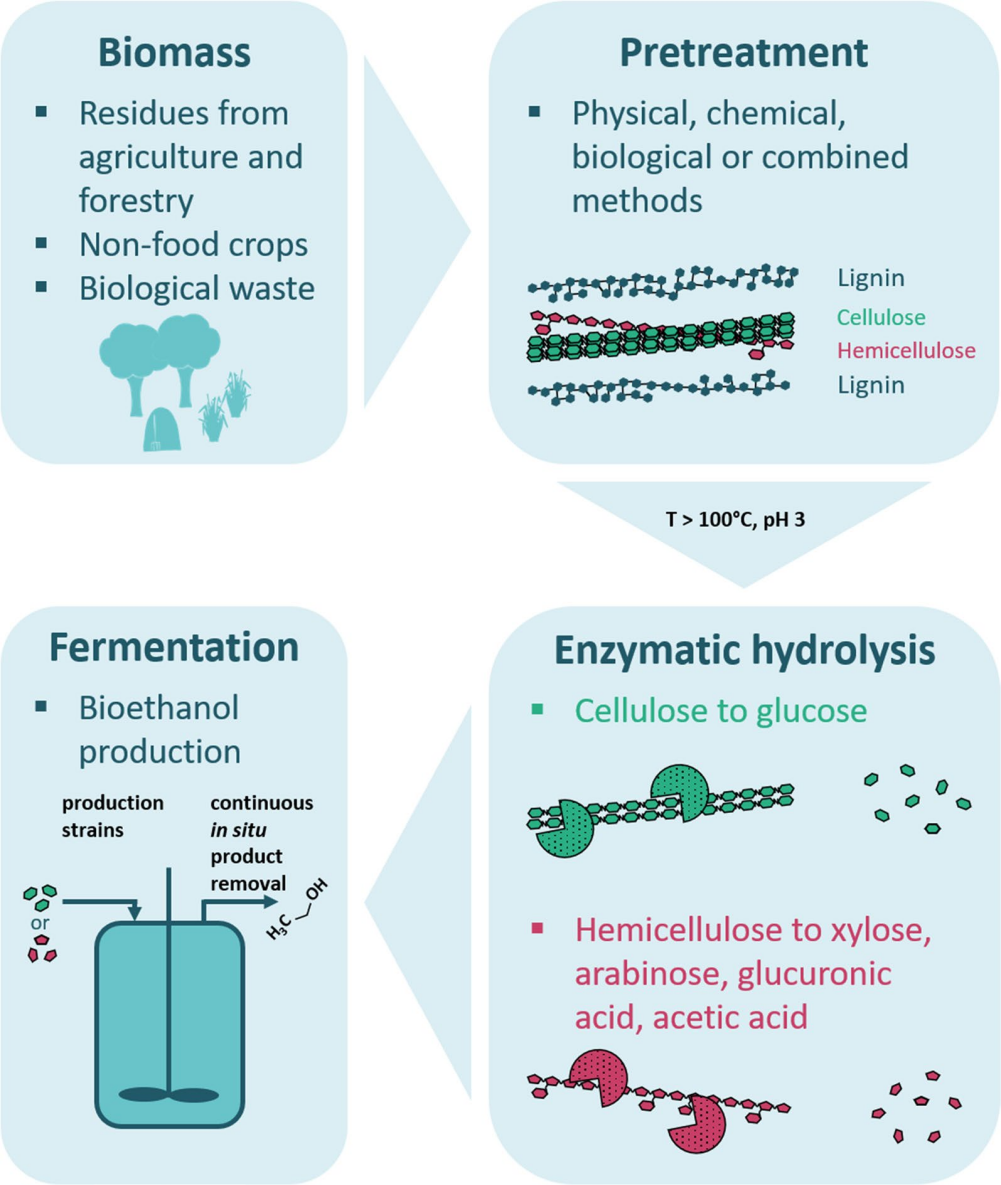
Application of plant-degrading hyperthermozymes in second-generation biorefinery

Fossil resources are still the main source of energy as well as for the production of many chemicals. To develop a sustainable economy without the use of these limited resources, governments worldwide initiated research and development strategies for the transition from an oil-based to a circular bio-based economy [1]. A central element of this bioeconomy is the development of sustainable biorefineries, which use renewable resources as feedstock, such as plant biomass, instead of oil [2] (Fig. 1).

The first generation of biofuels uses plant biomass from sugarcane, sugar beet, wheat and crops. Hence, first-generation biofuels, including bioethanol and biodiesel, are mainly produced from starch and vegetable oils [3, 4]. Nevertheless, since biomass for first-generation biofuels consists of potentially edible plant material and, further, requires large areas of agriculture fields, other sources of biomass had to be considered. This led to the development of the second generation of biofuels, which is based on lignocellulosic biomass. Lignocellulose consists of valuable polysaccharides, is abundant in agricultural residues and wood materials, and can be obtained from non-food feedstocks [5]. Despite these advantages, a major challenge is formed by the recalcitrant character of lignocellulose, which necessitates a pretreatment of this substrate for fractionation, for example, by combining physical and chemical pretreatment methods [6–8].

Efficient pretreatment results in the cleavage of lignocellulose enabling the enzymatic accessibility of its components: cellulose, hemicellulose and lignin. The first two components can be enzymatically hydrolyzed to yield hexose and pentose monomers, which can subsequently be fermented to ethanol or other alcohols and chemicals by anaerobic bacteria and fungi [9–14]. To save energy and avoid expensive cooling steps, combinatorial approaches for physicochemical biomass pretreatment with simultaneous enzymatic hydrolysis were developed [15, 16]. For this purpose, extremely heat-active and heat-stable GHs are needed. Since archaea have been significantly less studied than bacteria and eukaryotes, they present a so-far underexploited source of novel hyperthermozymes particularly useful for biorefineries [17].

Biorefinery concepts depend on the applied renewable resources including plant polymers such as cellulose, starch, xylan and mannan. Since these differ in the glycosidic linkages of their backbones, many different kinds of GHs are needed to hydrolyze these polysaccharides [17]. Therefore, integrated biorefinery processes need a variety of GHs, including cellulases, amylases, mannosidases and pullulanases, which are stable towards the respective process conditions. Many biotechnological processes are performed at elevated temperatures to improve the solubility and bioavailability of organic compounds and biomass [18]. Further benefits of high process temperatures include increased diffusion rates and a significantly reduced contamination risk [19, 20]. As a consequence, enzymes derived from hyperthermophilic microorganisms have become highly popular for such high-temperature industrial processes since hyperthermozymes exhibit maximum activity at temperatures around 100 °C and are extremely thermostable [21]. Furthermore, when simultaneously applying hyperthermozymes with different substrate specificities, but similar temperature and pH preferences, synergistic effects can be especially beneficial for the efficient utilization of plant biomass [22] (Fig. 1). In this review, we are highlighting the impressive characteristics of already characterized hyperthermozymes obtained from archaea, and summarize some interesting strategies to discover novel ones.

## Discovery of the potential of archaea and their glycoside hydrolases

The full potential of the extreme lifestyle at elevated temperatures was recognized in 1981, when Karl Stetter and Wolfram Zillig discovered life above a temperature of 80 °C with the isolation of the first hyperthermophilic species *Methanothermus fervidus*, which was unimaginable before [23, 24]. Later it was impressively shown that some hyperthermophiles are even growing at temperatures around (or above) 100 °C, like heterotrophic members of the genera *Pyrococcus* [25, 26] and *Thermococcus* [27], or the chemolithoautotroph *Pyrolobus fumarii* [28]. Beside the impact of these impressive findings for ecology [29] and evolution [30–33] and specially for our understanding of microbial physiology and metabolisms at extreme habitats [34–36], the discovery of hyperthermophiles also paved the path for the finding and characterization of extremely heat-stable biocatalysts, which are naturally produced by these microorganisms. While some hyperthermophiles, such as *Methanopyrus kandleri* are strictly chemolithoautotrophic [37], also heterotrophic hyperthermophilic representatives were isolated and characterized. Since a heterotrophic metabolism requires enzyme machineries which are able to degrade and utilize organic biomass, the use of such heterotrophic archaea or their recombinantly produced extremely heat-stable GHs was investigated for high-temperature industrial processes such as production of food, beverage, detergent and chemical products, as well as biomass pretreatment for biofuel generation [17, 38, 39]. Therefore, genes encoding GHs, such as amylases and xylanases, were cloned from isolated and known hyperthermophiles, produced (mostly in *E. coli*) and characterized. Interestingly, since some polysaccharide-degrading enzymes are secreted by their native organisms, biochemical characterizations of these extracellular enzymes revealed that their optimal activity can be found close to the temperature of the optimal growth rate of the respective microorganism (Table 1). Furthermore, besides the impressive heat activity, features of some archaeal plant-biomass-degrading enzymes revealed a concomitant high activity under acidic conditions and high pressure [40–42].

**Table 1:** Archaeal heterotrophic hyperthermophiles and characterized GHs

Species*	Growth range [°C]	*T*_opt_ [°C]	Characterized GH derived from organism**	*T*_opt_ [°C]	*T*_stab_	Genbank accession no.***
*Pyrococcus furiosus* [25]	70–103	100	Endoglucanase (GH12) extracellular enzyme [46] No Genbank	100	*T*_1/2_ 40 h at 95 °C	WP_011011985.1 (from CAZy website)
			Endoglucanase (GH16) extracellular enzyme [47]	105	100% of residual activity after incubation at 80 °C for 110 h	AF013169
			Amylase extracellular enzyme [44]	100	> 60% relative activity at 120 °C after incubation for 1 h	n.l.
			Amylase intra- and extracellular enzymes [43]	106	30% residual activity after incubation for 8 h at 98 °C	n.l.
			Amylase extracellular enzyme [48]	100	80% residual activity after incubation for 6 h at 100 °C	U96622
			Amylase intracellular enzyme [49]	100	> 80% residual activity after incubation for 2 h at 100 °C	n.l.
			Glucosidase intracellular enzyme [50]	102–105	*T*_1/2_ 85 h at 100 °C	n.l.
			Glucosidase secretion n.l. [51]	100	80% residual activity after incubation for 1 h at 100 °C	AF013169
*Pyrococcus horikoshii* [26]	< 80–102	98	Endoglucanase (GH5) extracellular enzyme [52]	97	Relative activity 80% after incubation at 97 °C for 3 h	AAQ31833.1 (from CAZy website)
*Pyrococcus woesei* [53]	n.l.	100–103	Amylase extracellular enzyme [54]	100	No loss of activity after incubation at 90 °C for 6 h	n.l.
*Palaeococcus pacificus* [55]	50–90	80	Cyclodextrinase PpCD (GH13) secretion n.l. [56]	95	> 90% relative activity after 8 h at 85 °C	WP_048164969
*Thermofilum pendens* [57]	85–90	Up to 95	Glucosidase (GH 3) intracellular enzyme [51]	90	50% residual activity after incubation for 60 min at 95 °C	YP_920894
*Staphylothermus marinus* [58]	n.l.	98	Amylase SMMA (GH13) secretion n.l. [59]	100	Melting point *T*_m_ 109 °C	WP_011838911.1
*Thermococcus chitonophagus* [60]	60–93	85	Chitinase chi70 associated with outer cell membrane [61]	70	> 50% activity after incubation at 120 °C for 1 h	n.l.
*Thermococcus kodakarensis* [62]	60–100	85	Pullulanase TK-PUL (GH13) secretion n.l. [63]	95–100	T_1/2_ 45 min at 100 °C	BAD85166.1
*Saccharolobus shibatae* [64]	Up to 86	81	Endoglucanase (GH 12) secretion n.l. [65]	100	100% activity after incubation at 85 °C for 1 h	LT221867
*Sulfolobus acidocaldarius* [66]	55–80	70–77	Amylase (GH 57) membrane-bound enzyme [67]	105	*T*_1/2_ 2.5 h at 100 °C	AAY80509.1 (from CAZy website)
*Saccharolobus solfataricus* [68]	n.l.	70	Xylanase membrane-bound enzyme [69]	90	T_1/2_ 47 min at 100 °C	n.l.
			Endoglucanase SSO1949 (GH 12) extracellular enzyme [41]	80	*T*_1/2_ 8 h at 80 °C (pH 1.8)	AAK42142.1; NP_343352.1 (from CAZy website)
			Galactosidase intracellular enzyme [70]	95	> 3 h at 75 °C	n.l.
			Galactosidase secretion n.l. [71]	90	> 2 h at 75 °C	n.I.
			Galactosidase (GH36) intracellular enzyme [72]	90	*T*_1/2_ 30 min at 90 °C	n.l.
			Xylosidase (GH 31) secretion n.l. [73]	90	*T*_1/2_ 38 h at 90 °C	AJ251975
			Mannosidase (GH 38) intracellular enzyme [74]	85	> 20% activity after 10 min at 80 °C	AAK43108.1; AIX48014.1; CAC24028.1; NP_344318.1 (from CAZy website)
*Caldivirga maquilingensis* [75]	60–92	85	Galactosidase (GH1) secretion n.l. [76]	110	100% relative activity after 120 min at 90 °C	ABW01253.1
*Pyrobaculum aerophilum* [77]	75–104	100	Glucosidase (GH31) intracellular [78]	90	46% relative activity after 1 h at 110 °C	gi:18160499
*Picrophilus torridus* [79]	45–65	60	Glucosidase (GH31) intracellular enzyme [80]	87	81% relative activity after 120 min at 80 °C	AAT42677.1 (from CAZy website)
			Mannosidase (GH38) intracellular enzyme [80]	70	50% relative activity after 1 h at 80 °C (with Cd^2+)^	AAT42676.1

Functional screening of metagenomes derived from hydrothermal systems						
Metagenome			Characterized GH derived from metagenome (archaeal origin)	*T*_opt_ [°C]	*T*_stab_	Genbank accession no.***
Deep sea hydrothermal vent			Endoglucanase (GH 12) extracellular enzyme [81]	92	> 80% activity after incubation at 80 °C for 4,5 h	LN850140 (fosmid insert)
Hot spring			Glucosidase (GH 1) secretion n.l. [40]	90	> 67% activity after incubation at 90 °C for 1.5 h	HG326254

Combinatorial approach of enrichment culture and screening						
Enrichment culture*	Archaeon		Characterized GH derived from enrichment culture	*T*_opt_ [°C]	*T*_stab_	Genbank accession no.***
Shallow marine vent sample from Vulcano Island, incubation 90 °C, cellulosic substrate, anoxic [82]	Unknown, not cultivated in pure culture, probably related to *Thermococcus*		Endoglucanase Vul_Cel5A (GH 5) extracellular enzyme [42]	115	*T*_1/2_ 43 min at 100 °C	MH910342
			Glucosidase (GH 1) intracellular enzyme [22]	105	*T*_1/2_ 99 min at 75 °C	MN329095
Sample from 94 °C geothermal pool of northern Nevada, incubated at 90 °C, cellulosic substrates, anoxic [83]	Unknown, not cultivated in pure culture, probably related to *Ignisphaera*		Endoglucanase EBI-244 (multidomain) extracellular enzyme [83]	109	*T*_1/2_ 4.5 h at 100 °C	JF509452
*In situ* enrichment culture in hot vent (76–99 °C, Kuril archipelago) using xylan as carbon source [84]	*Thermococcus* sp. Strain 2319X1		Multidomain glycosidase MDG (Multidomain) extracellular enzyme Full-length protein Truncated GH5 version [84]	60 90	n.l. n.l.	CP012200 (genome)

Selection criteria of the reported GHs shown in the table are a high thermo-activity and thermo-stability. GH families (in brackets) and Genbank accession numbers are provided if the respective articles contain this information

*n.l.* not listed in the original paper

* Literature source reports the first description of the organism

** Literature source reports the enzyme characterization

*** As provided by the literature source or CAZy

**Fig. 2:**
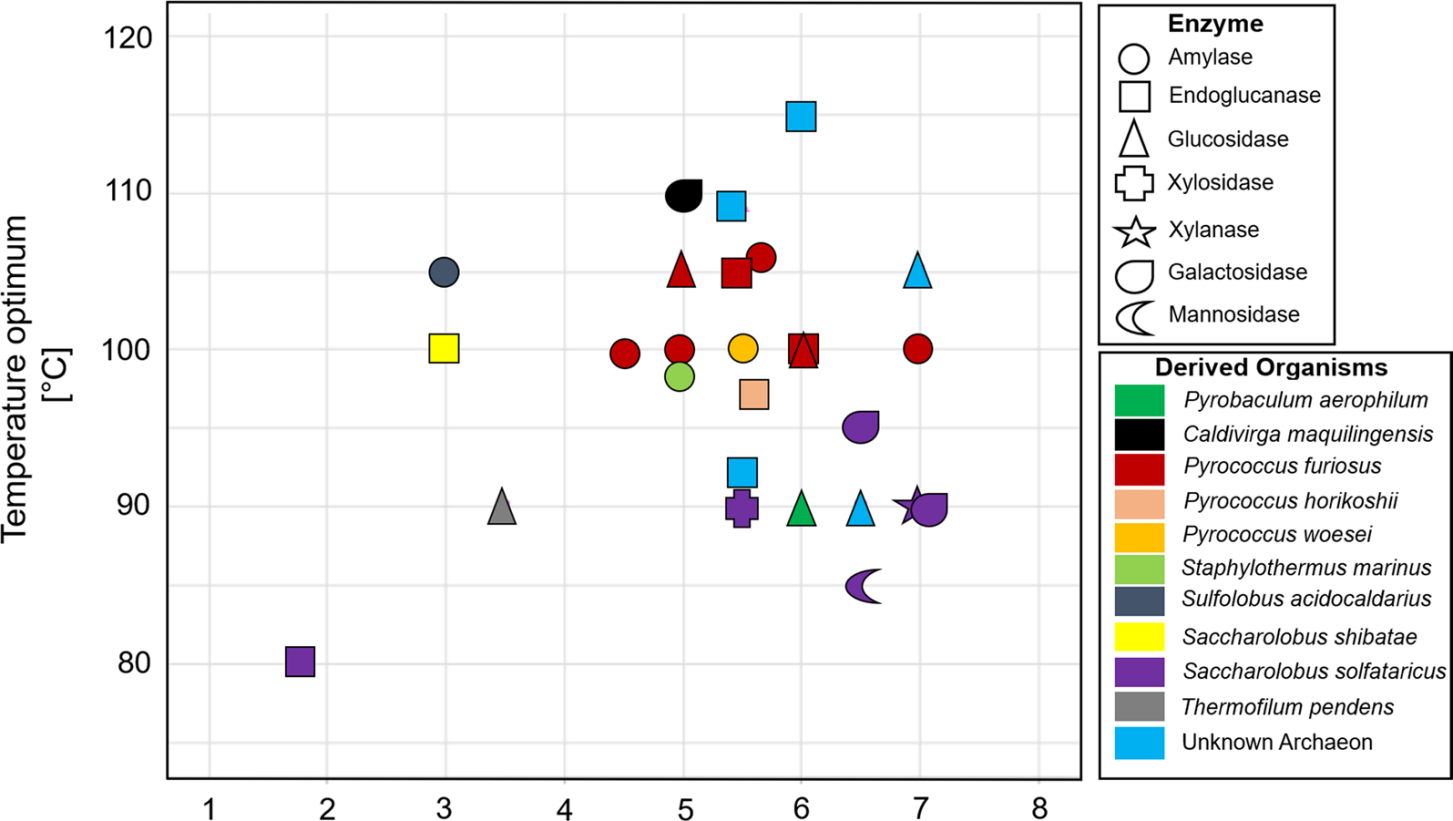
Biochemical characteristics of known biomass-degrading GHs of archaeal origin. The following GHs were included in the figure: endoglucanases of *Pyrococcus furiosus* [46, 47], *Pyrococcus horikoshii* [52], *Saccharolobus shibatae* [65], *Saccharolobus solfataricus* [41], and three unknown archaea [42, 81, 83]; amylases of *Pyrococcus furiosus* [43, 44, 48, 49], *Sulfolobus acidocaldarius* [67], *Pyrococcus woesei* [54], and *Staphylothermus marinus* [59]; sylanase of *Saccharolobus solfataricus* [69]; glucosidases of *Pyrococcus furiosus* [50, 51], *Pyrobaculum aerophilum* [78] and two unknown archaea [22, 40]; galactosidases of *Saccharolobus solfataricus* [70, 71] and *Caldivirga maquilingensis* [76]; xylosidase of *Saccharolobus solfataricus* [73]; mannosidase of *Saccharolobus solfataricus* [74]

Within the last 30 years, numerous GHs of already isolated hyperthermophiles were produced using sequence- or function-based screening methods (Table 1). Among the first-characterized GHs were amylases from the hyperthermophile *Pyrococcus furiosus*, which were obtained by performing cultivations on carbohydrates with subsequent GH detection and activity tests of the crude cell extract as well as the supernatant of the culture. The detected and characterized amylases exhibited maximum activity at temperatures higher than 99 °C [43, 44] (Table 1). Within the last decades, more GHs of *Pyrococcus furiosus* were discovered and characterized, including endoglucanases, amylases and glucosidases, which are all working optimally at temperatures around 100 °C (Table 1). Additionally, numerous characterized archaeal GHs, including endoglucanases, amylases, mannosidases and glucosidases, were characterized from pure strains of *Saccharolobus solfataricus* (previously *Sulfolobus solfataricus* [45]) *Saccharolobus shibatae* (previously *Sulfolobus shibatae* [45]), *Pyrococcus horikoshii*, *Pyrococcus woesei*, *Sulfolobus acidocaldarius*, *Staphylothermus marinus* and *Thermofilum pendens* (Table 1, Fig. 2). However, due to the fact that cultivation of new hyperthermophiles in pure cultures is most often a challenging task, many thermozymes and some hyperthermozymes were detected using culture-independent metagenomic approaches.

## Classification of GHs demonstrates a small number of characterized archaeal enzymes

GHs are classified based on primary sequences (Carbohydrate-Active enZyme database CAZy, www.cazy.org) or function (Enzyme Commission EC, established in 1992) [85]. The amino acid structure-based classification of CAZy is an adequate way to predict and identify mechanisms and specificities of glycoside hydrolases (GHs) [86, 87]. So far, CAZy divides GHs into 167 GH families, and the GH 5, 13, 16, 30 and 43 families are again divided into several subfamilies [88–92] (www.cazy.org). GH family 5 represents one of the largest GH families and consists of 16,520 enzymes as noted in June 2020. The deposited primary protein sequences of GH family 5 demonstrate the lack of knowledge about archaeal GH since 99 archaeal protein sequences are vastly outnumbered by 13,137 bacterial ones. From these deposited sequences, 5 archaeal GHs are listed as characterized (two cellulases and three endoglucanases), compared to 559 bacterial ones. Regarding the activity and function of the structurally related proteins of GH5, cellulolytic and hemicellulolytic enzymes are present, including endo-β-1,4-glucanases (EC 3.2.1.4), β-glucosidases (EC 3.2.1.21), licheninases (EC 3.2.1.73), and endo-β-1,4-mannosidases (EC 3.2.1.78), and are, therefore, of industrial interest. GH5 subfamily 1 contains the extremely stable endoglucanase of *Pyrococcus horikoshii* [52], whose structure was analyzed via X-ray crystallography in 2008 [93, 94] confirming the typical GH5 (β/α)_8_ fold. In a recent study of Strazzulli and colleagues, a novel archaeal mannanase was discovered, belonging to the subfamily GH5_19 [95].

One explanation for the imbalance of archaeal:bacterial GHs is probably the cultivation challenge that comes along with many archaeal species, which are mainly found in extreme habitats in terms of pH, salinity and temperature and pressure. However, within the last years, the distribution of archaeal species was focused in the research field of microbial ecology [96], and numerous studies detected, with cultivation-independent methods, a distribution of archaea in non-extreme environments, such as sulfur-rich lakes [97], marine sediments and water columns [98, 99], estuarine ecosystems [100] or the grass-root zone [101]. Biotechnology will benefit from this increasing knowledge of archaeal ecology since more and more binned genomes of uncultivated archaea and novel Candidatus species will be published that can be used for enzyme screening. To date, 367 genomes (CAZomes) of archaea are published in CAZy, compared to 17,054 bacterial ones. The listed genomes of CAZy of heterotrophic archaea are exhibiting diverse GH families with interest of various biotechnological disciplines. For example, the archaeon *Staphylothermus marinus* (Taxonomy ID 399550) contains a total number of 11 GHs of family 1, 4, 13, 38, 57, 84, and 122 and, therefore, offers potentially heat-stable glucosidases, galactosidases, amylases, mannosidases and glucosaminidases. One additional challenge of understanding and making use of archaeal GH machineries lies in the whole metabolism of archaea, which is considered to be a complex “mixture” of bacterial- and eukaryotic-like pathways resulting in modified pathways [102–104]. Studies focusing on transcriptomics of cultured archaea or reassembling of uncultured archaeal genomes will provide highly useful insights into new archaeal metabolisms, and novel catabolism reactions could be investigated for degradation of complex substrates [105].

## Culture-dependent approaches coupled with metagenomics for the identification of promising extremozymes

The revolution of enzyme discovery and microbial diversity analysis came along with the next-generation sequencing (NGS) technology, which is based on the fragmentation of (meta)genomic DNA, followed by sequencing of the resulting fragments, thus allowing millions of (high-throughput) sequencing reactions in parallel [106]. With sinking costs for NGS, this technology became the gold standard in all areas of life sciences, reaching from the analysis of human microbiome consortia [107] to microbial communities in extreme hydrothermal ecosystems [108]. Furthermore, this method allowed a deeper insight, not just into genera abundance of the composition of microbial communities, but also into the metabolic pathways of these consortia [109]. Bioinformatic tools including MG-RAST [110] and MEGAN [111, 112] were developed as valuable means for calculating the taxonomy of metagenomes based on known sequences of a database (reference-based classification). Since metagenomic sequences cannot only be used for diversity analysis, but also for identification of putative carbohydrate-encoding genes, the metagenomic era supported the detection of a huge number of novel enzyme candidates [113].

In addition, it was shown that interdisciplinary approaches, consisting of microbial diversity analyses and screening for novel GH-encoding genes, are a promising combination which leads to successful identification of novel enzymes, in particular when analyzing extreme habitats such as hydrothermal systems [83, 95, 114]. Environmental samples of extremely hot ecosystems contain a huge variety of microorganisms with different metabolisms, ranging from chemolithoautotrophy to heterotrophy, and the pool of coding sequences in such a sample is huge in relation to the extreme conditions of such an environment. Thus, to focus on selected target enzymes, enrichment cultures with conditions that support only the microbes with metabolisms of interest can be applied, and, therefore, select a defined microbial pool [115]. In the following, we will focus on three specific archaeal plant-biomass degrading enzymes that were detected by performing culture-dependent approaches:

The research team of Frank Robb aimed to gain novel insights into archaeal degradation of crystalline cellulose, which still is a very unexploited field of research [83]. The team performed strictly anaerobic enrichment cultures at a temperature of 90 °C using sediment samples from a terrestrial geothermal source of Nevada. Enrichment cultures were transferred several times into fresh medium and microcrystalline cellulose and Whatman filter paper were used as carbon and energy source. Using this approach, a three-species consortium was enriched, whose 16S rRNA genes showed highest similarities to the archaeal genera *Ignisphaera*, *Pyrobaculum* and *Thermofilum*. Using a metagenomic approach, cellulase-encoding genes of GH family 5 were screened and one predicted GH, labeled as EBI-244, represented a potential multidomain cellulase. This cellulase seemed to consist of four structural domains, and while one of these domains showed similarities to GH5, the three remaining domains did not show any similarity to known GH families. Characterization of the heterologously produced protein in *E. coli* revealed highly impressive characteristics: a temperature optimum of 109 °C, a temperature melting point of 113 °C, a half-life time of 5 h at 100 °C, as well as resistance against ionic liquids, detergents and salts, and finally, a high activity towards crystalline cellulose (Avicel). The unique composition of the different domains of this enzyme proved to be interesting, and furthermore, the catalytic domain and the whole sequence showed high similarities to non-thermophilic eukaryotic mannanases.

Another combinatorial approach linking cultivation and genomics for the identification of novel archaeal plant-degrading enzymes was used by the team of Bettina Siebers [84]. An in situ enrichment culture was performed in a hot vent of the Kuril archipelago using birchwood xylan as substrate. This sophisticated experimental setup resulted in the successful isolation of *Thermococcus* sp. strain 2319X1, which is able to grow on xylan as sole carbon and energy source. By performing genome sequencing and genome reassembly of this specific strain, a multi-domain glycosidase (MDG) was detected. The protein contains three GH domains, one of GH family 5 and two of GH family 12, which could explain the impressive multifunctionality towards a broad substrate spectrum, including Avicel, carboxymethyl cellulose, β-1,4 linked and β-1,3 linked glucose polysaccharides, as well as xylose-based and mannose-based carbohydrates. The enzyme showed, in contrast to most endoglucanases of archaeal origin, optimal activity in alkaline milieu (optimum pH 8.5).

A third example for a combinatorial and multidisciplinary approach in regard to cultivation and omics technologies for archaeal enzyme discovery was performed using samples of the extremely shallow marine vents of Vulcano Island, which were taken at a temperature of 100 °C [22, 42, 82]. Enrichment cultures of samples were performed under anoxic conditions at a temperature of 90 °C, using carboxymethyl cellulose as carbon source, and the diversity of the enrichment culture was analyzed using a metagenomic approach. The diversity analysis performed with MEGAN6 revealed that the community consisted of more than 96% of archaeal microorganisms with the hyperthermophilic genera *Thermococcus* and *Palaeococcus* showing the highest abundance in these cultures [82]. Afterwards, the metagenome was used for a sequence-based screening for GH5 endoglucanases, and the putative endoglucanase Vul_Cel5A was detected, which showed highest similarity to a putative endoglucanase of *Thermococcus* sp. and 56% identity to the characterized endoglucanase of *Pyrococcus furiosus* [42]. Production and characterization of the recombinantly produced enzyme Vul_Cel5A revealed impressive characteristics in regard to thermo-activity (T_opt_ of 115 °C), thermo-stability (T_1/2_ of 43 min at 100 °C) and resistance towards a broad range of detergents, as well as an extremely high relative activity and stability under acidic conditions. In addition, the metagenome was binned using Maxbin [116], which resulted in a reassembly of four genomes. Further genes encoding putative biomass-degrading enzymes were identified in the partially reassembled genome in which Vul_Cel5A was located. Cloning of the genes and production of the respective enzymes resulted in the identification and characterization of the archaeal β-glucosidase Vul_Bgl1A, which exhibited highest activity at 105 °C towards 4-nitrophenyl β-d-glucopyranoside and cellobiose [22]. Interestingly, by simultaneously applying Vul_Cel5A and Vul_Bgl1A, a significant increase of glucose formation was monitored indicating synergistic effects of the two enzymes.

All three combinatorial approaches highlight the importance of combining cultivation with state-of-the-art (meta-)genomic analyses to identify novel archaeal enzyme candidates. The detected and produced enzymes are highly promising candidates for industrial processes since all three enzymes exhibited, besides their high thermo-activity, a very broad biomass substrate spectrum and a relatively high activity in acidic or alkaline milieu. Regarding the sequence composition of the mentioned archaeal GH5 enzymes, xylanase MDG [84] showed a sequence identity of 46% with endoglucanase Vul_Cel5A [42], while the similarity of EBI-244 [83] to these enzymes was very low (23% to MDG and 19% to Vul_Cel5A). Improved combinatorial approaches, such as metatranscriptomic- and proteomic-based screening coupled with prior high-temperature cultivation on plant biomass, will probably have a high impact on prospective identification of novel hyperthermozymes for application in various biotechnological processes including biorefineries. The successful application of such a combinatorial approach was recently described by Zayulina and colleagues, who coupled cultivation of a novel archaeon *Thermofilum adornatum* with proteomic analyses to identify four novel cellulolytic enzymes [117].

## Protein engineering to tailor plant-degrading enzymes for industrial processes

While the implementation of plant-degrading hyperthermozymes in integrated biorefineries is a straightforward application of these robust biocatalysts, even these naturally already thermo-active and thermo-stable enzymes need further improvement before being subjected to the biorefinery process (Fig. 1) [1, 118]. In general, wild-type enzymes are not directly suitable for industrial application but have to be modified prior to usage in biotechnological processes due to oxidation sensitivity and generally low activities of the native enzymes. The replacement of oxidation-sensitive methionine residues is performed by site-directed mutagenesis, whereas improvements of substrate specificity and activity are gained by various rounds of protein-engineering coupled with screening for desired activities and/or stabilities [119, 120]. Applied protein engineering approaches need different levels of previous knowledge of the target enzyme, ranging from directed evolution applying random mutagenesis, which requires only the DNA sequence, to rational protein design, such as site-directed mutagenesis, which relies on the X-ray structure of the target enzyme. Nowadays, combinations of directed evolution and rational protein design are frequently applied with the aim to gain maximum benefit from each of the protein engineering techniques [121, 122].

In particular, rational protein-engineering methods have been successfully applied for improving plant-degrading enzymes since X-ray structures of hyperthermozymes are often being resolved and analyzed with the aim to understand the mechanisms that lead to their superior stability properties [123, 124]. One example of a promising plant-degrading hyperthermozyme with potential for further optimization is a β-glycosidase from the extreme thermoacidophilic archaeon *Saccharolobus solfataricus*. This hyperthermozyme was reported to exhibit maximal activity above 95 °C and remarkable stability towards detergents [125]. However, alkaline pH values seemed to have a strong destabilizing effect on this archaeal GH, which belongs to GH family 1 [126]. It was, therefore, concluded that the enzyme’s stability resulted from ionic interactions on the surface, which would be perturbated at alkaline pH [125].

However, the β-glycosidase from *Saccharolobus solfataricus* proved to be an excellent example for the successful heterologous production of an archaeal plant-degrading enzyme applying a yeast expression system [127]. The application of mesophilic *Saccharomyces cerevisiae* as host for industrial-scale fermentation enabled a fast and efficient purification strategy of the target enzyme by taking advantage of its exceptional heat stability when applying a heat precipitation of the host proteins [127]. The reasons for the enzyme’s heat stability were analyzed by creating mutants of the hyperthermozyme, which, for example, hampered the formation of an ion pair network at the tetrameric interface of the enzyme and led to heat-sensitive mutants [128]. These results further supported the current hypothesis that ionic interactions are of major importance for protein stability of hyperthermophiles. Furthermore, when comparing the enzyme to β-glycosidases from *Thermosphaera* and *Pyrococcus furiosus*, it was deduced that oligomerization could be another general factor for protecting hyperthermozymes from degradation [128–130]. General mechanisms of protein unfolding were analyzed by creating mutants of the N and C terminii of the β-glycosidase from *Saccharolobus solfataricus* [128, 131]. The respective studies showed that the quaternary structure was crucial for stability of this hyperthermozyme [128] and that fraying of the polypeptide chain termini played an important role in protein unfolding [131].

In addition to rational approaches, also random mutagenesis studies involving suitable in vivo selection mechanisms were conducted with hyperthermozymes. One study focusing on the β-glycosidase from *Saccharolobus solfataricus* showed that mutations far from the active site may have crucial impact on the enzyme’s activity and stability profiles [132]. While a mutant with three random mutations showed a twofold enhanced specific activity towards galactosides at 85 °C, the higher flexibility of the enzyme variant that enabled this increase in substrate turnover also led to an almost 300-fold reduced thermal stability. Directed evolution was also applied in a study focusing on broadening the temperature profile of a β-glucosidase from the hyperthermophilic archaeon *Pyrococcus furiosus*. Here, the low-temperature activity of the hyperthermozyme was significantly increased with twofold enhanced activity towards cellobiose at 20 °C [133].

Successful engineering of another hyperthermozyme was previously demonstrated by Kang et al. when further improving the thermo-active and thermo-stable cellulase from *Pyrococcus horikoshii* [52, 134]. In this study, rational protein design was performed by eliminating cysteine residues and adding a carbohydrate-binding domain to increase the cellulase’s activity. The successful approach led to the remarkable observation that the thermo-stability of the enzyme was not significantly impaired by removing disulfide bonds. Furthermore, increased affinity towards crystalline cellulose was obtained by the addition of a chitin-binding domain from another hyperthermozyme, leading to the conclusion that the generation of sophisticated fusion proteins might be a suitable means to tailor hyperthermozymes for industrial application [134].

Despite the fact that hyperthermozymes offer a huge potential for industrial application, there are only few examples of actual utilization of these enzymes. This is mainly due to the fact that in contrast to their mesophilic counterparts, a significantly lower number of hyperthermozymes is known to date. Furthermore, they are often more difficult to produce at high amounts as there are no industrially approved extremophilic production strains available yet. However, it was shown that expression problems with mesophilic hosts, such as *E. coli*, might be overcome by designing synthetic genes, which was successfully applied for a phosphopantetheine adenylyltransferase from the hyperthermophilic archaeon *Pyrococcus abyssi* [135]. In a different approach, careful adjustment of expression conditions was sufficient to produce an archaeal chitinases with *E. coli* expression strains [136].

Another example highlighting the potential of hyperthermozymes for industrial application is the development of a continuous process for lactulose production by implementation of immobilized thermostable β-glycosidase from *Pyrococcus furiosus* [137]. With the advance of more thorough analyses of archaea, including the Archaeal Proteome Project (ArcPP), further insights into the mechanisms and beneficial properties of archaeal enzymes are expected in the near future [102, 138].

## Conclusion

Characterizations of already known archaeal thermo-active and thermo-stable biomass-degrading GHs have highlighted their potential for high-temperature industrial processes. Archaeal GH properties provide interesting features for an efficient biomass conversion and biofuel generation. To discover new promising candidates, it is necessary to study in detail the microbiology, physiology and enzymology of microorganisms of extremely hot habitats, and to combine and implement this generated knowledge for an efficient screening for novel promising GH candidates. Combinatorial approaches of cultivation and omics technologies lead to the discovery of highly interesting archaeal GHs with outstanding characteristics. Current and future global challenges require sustainable biobased solutions, and bioeconomy is becoming an important field to meet these challenges. Still, one of the major challenges is the efficient transformation of recalcitrant plant biomass to polysaccharides that can be used as a resource for countless fermentation processes. Archaeal hyperthermozymes represent an ideal platform to support this crucial step in an eco-friendly way.

## Data Availability

Not applicable.

## Abbreviations

GH: glycoside hydrolases
CBM: carbohydrate-binding modules
